# The linear framework II: using graph theory to analyse the transient regime of Markov processes

**DOI:** 10.3389/fcell.2023.1233808

**Published:** 2023-11-03

**Authors:** Kee-Myoung Nam, Jeremy Gunawardena

**Affiliations:** Department of Systems Biology, Harvard Medical School, Boston, MA, United States

**Keywords:** linear framework, graph theory, Matrix-Tree theorems, rational functions, Markov processes, first-passage times

## Abstract

The linear framework uses finite, directed graphs with labelled edges to model biomolecular systems. Graph vertices represent chemical species or molecular states, edges represent reactions or transitions and edge labels represent rates that also describe how the system is interacting with its environment. The present paper is a sequel to a recent review of the framework that focussed on how graph-theoretic methods give insight into steady states as rational algebraic functions of the edge labels. Here, we focus on the transient regime for systems that correspond to continuous-time Markov processes. In this case, the graph specifies the infinitesimal generator of the process. We show how the moments of the first-passage time distribution, and related quantities, such as splitting probabilities and conditional first-passage times, can also be expressed as rational algebraic functions of the labels. This capability is timely, as new experimental methods are finally giving access to the transient dynamic regime and revealing the computations and information processing that occur before a steady state is reached. We illustrate the concepts, methods and formulas through examples and show how the results may be used to illuminate previous findings in the literature.

## 1 Introduction

The linear framework is a graph-theoretic approach to analysing biomolecular systems (Gunawardena, 2012; Mirzaev and Gunawardena, 2013; Gunawardena, 2014). A recent review (Nam et al., 2022) described how the framework has been used to study systems at steady state, in contexts such as post-translational modification and gene regulation. The present paper is a sequel to this review, which describes how the graph-theoretic approach can be extended to the transient regime, prior to the steady state being reached, for systems that are Markov processes. These new results were introduced in the first author’s Ph.D. thesis (Nam, 2021) and full details with complete proofs are being published separately (Nam and Gunawardena, 2023). The purpose of the present paper is to provide an elementary introduction to this circle of ideas for a wider readership in cell and developmental biology. We hope this will be of interest to anyone who wants to explore the transient regime for biological systems that can be modelled by Markov processes.

**FIGURE 1:**
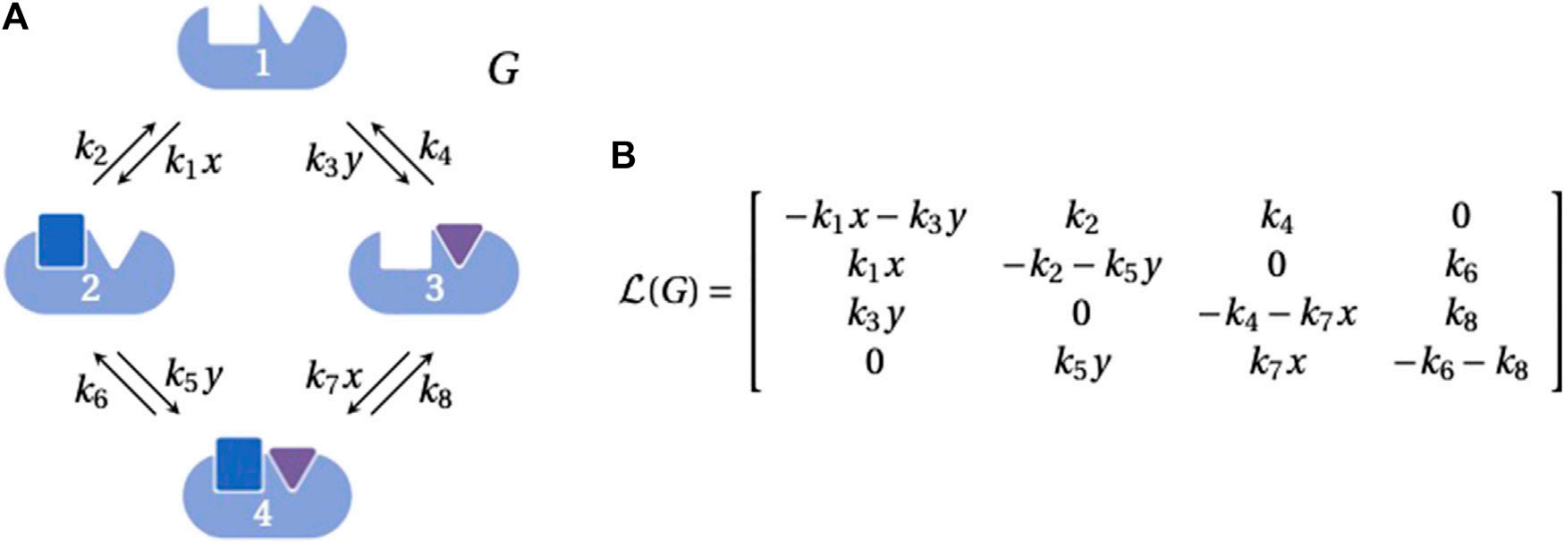
Linear framework graph and Laplacian matrix. **(A)** An example graph, *G*, representing the binding of two ligands, each to one site, on a biomolecule, with vertices indexed 1, *…*, 4 as shown. The labels on the edges 1 → 2 and 3 → 4 include the concentration, *x*, of the blue ligand that binds to the first site and the labels on the edges 1 → 3 and 2 → 4 include the concentration, *y*, of the purple ligand that binds to the second site. The parameters *k*_1_, *k*_3_, *k*_5_ and *k*_7_ are on-rates for binding, with dimensions of (concentration × time)^−1^; the other parameters are simple rates with dimensions of (time)^−1^. Graphics were generated using BioRender.com. **(B)** The Laplacian matrix, L(G) , for the graph in panel **A**.

Linear framework graphs (hereafter, “graphs”) are finite, simple, directed graphs with labelled edges. (A simple graph is one in which there is at most one edge between any two distinct vertices and there are no self-loops.) Graph vertices, usually denoted 1, 2, 3, *…*, represent chemical species or molecular states; edges, denoted *i* → *j*, represent reactions or transitions; and edge labels, denoted *ℓ*(*i* → *j*), represent rates which are positive and have dimensions of (time)^−1^. Importantly, the labels may include expressions that describe how the underlying system is interacting with its environment. For example, the graph in Figure 1A shows how ligand binding gives rise to concentration terms in the edge labels.

A graph yields a linear dynamics, from which the linear framework gets its name. The dynamics is most simply described by imagining that the edges are chemical reactions with the edge labels as the rate constants for mass-action kinetics. Since each reaction has only a single substrate, the resulting dynamics is necessarily linear and can be expressed in matrix form as

$$\frac{du\left(t\right)}{dt}=L\left(G\right)\cdot u\left(t\right).$$ (1)

Here, $u\left(t\right)={\left(u_{1}\left(t\right),\ldots ,u_{N}\left(t\right)\right)}^{T}$ is the column vector of concentrations at each of the *N* vertices, and L(G) is the Laplacian matrix of the graph (Figure 1B). Graph Laplacians are defined with varying conventions and scalings and they may be interpreted as discrete versions of the classical Laplacian differential operator (Chung, 1997). From this viewpoint, Eq. 1 is a discretised diffusion equation. Since matter is neither created nor destroyed during the dynamics, there is a conservation law,

$$u_{1}\left(t\right)+\cdots +u_{N}\left(t\right)=u_{\text{tot}}.$$ (2)

Eq. 2 manifests itself in the column sums of the Laplacian being zero, 1⋅L(G)=0 (Figure 1B), where 1 denotes the all-ones row vector of the appropriate dimension.

The framework is typically used in two contexts: for bulk biochemistry of reacting chemical species, where *u*(*t*) in Eq. 1 describes the deterministic time evolution of species concentrations; and for individual molecular systems that exhibit stochastic transitions, where *u*(*t*) describes the deterministic time evolution of the probabilities of the molecular states. In the latter case, since probabilities sum to 1, *u*_tot_ = 1. It is interesting that the same mathematics describes both contexts. Here, we will be working in the context of individual molecules and stochastic transitions. From now on, *u*(*t*) will be the vector of probabilities and we will assume that *u*_tot_ = 1.

The graph formulation allows nonlinear biochemistry, which often arises from ligand binding, to be disentangled into a linear part carried by the linear dynamics in Eq. 1 and a nonlinear part that comes through the edge labels (Nam et al., 2022). The terms appearing in the labels, such as ligand concentrations (Figure 1A), have to be dealt with separately. They may be specified by separate conservation laws or by other graphs (Nam et al., 2022). For the present paper, we will assume that any ligands that are interacting with a graph are present in “reservoirs” (Nam et al., 2022, §4), similar to thermodynamic reservoirs, so that their free concentrations do not change upon binding. Accordingly, edge labels are treated as constants over the timescale of the dynamics in Eq. 1. In this case, for the stochastic context described above, the graph specifies the infinitesimal generator for a finite-state, continuous-time, time-homogeneous Markov process, *X*(*t*), (hereafter, a “Markov process”), so that the edge labels are given by,

$$\ell \left(i\to j\right)=\underset{h\to 0}{\lim}\frac{\mathrm{Pr}\left(X\left(t+h\right)=j|X\left(t\right)=i\right)}{h},$$

whenever the right-hand side is nonzero and therefore positive. (A zero infinitesimal rate does not yield an edge.) Conversely, any such Markov process with an infinitesimal generator is specified by a graph (Mirzaev and Gunawardena, 2013, Theorem 4). The Laplacian dynamics in Eq. 1, with *u*_tot_ = 1, becomes the master equation for the forward evolution of the vertex probabilities, *u*(*t*). The linearity of the linear framework is perhaps less surprising now, as master equations are, indeed, linear (van Kampen, 1992). We see that, within reservoir assumptions, the linear framework provides a graph-theoretic way to define and study the Markov processes that have been widely used to model biological systems.

Surprisingly, the graph rarely makes an appearance in the Markov process literature. This may be because the graph theory has so far primarily been used to study steady states of the Laplacian dynamics (Nam et al., 2022), which may not have been of much mathematical interest outside of applications in biology. Since Eq. 1 is linear, it can readily be solved in terms of the eigenvalues and eigenvectors of L(G) . Recall that if L(G)⋅v=λv , for some vector *v* and some scalar *λ*, then *v* is an eigenvector for the eigenvalue *λ* (Strang, 2022). By definition, the steady state of Eq. 1, which we will denote by *u* ^*∞*^(*G*), satisfies *du* ^*∞*^(*G*)/*dt* = 0, so it follows from Eq. 1 that $L\left(G\right)\cdot u^{\infty }\left(G\right)=0$ . In other words, *u* ^*∞*^(*G*) is an eigenvector for the zero eigenvalue.

When *G* is *strongly connected* (see below), the steady state, *u* ^*∞*^(*G*) is unique. This particular eigenvector can be calculated from L(G) using the determinants of principal sub-matrices, or the *first minors* of L(G) , which thereby have terms of alternating sign (Strang, 2022). It is a remarkable property of Laplacian matrices that extensive cancellations take place so that their minors can be written as *manifestly positive polynomials* in the edge labels (Eq. 5). A polynomial is a sum of *monomials*, where a monomial is an algebraic expression consisting solely of a product of variables and a numerical coefficient, like 5*a* ^3^ *bc* ^2^ (Barbeau, 1989). A polynomial is manifestly positive if the numerical coefficient of each monomial is positive. (A polynomial like *a* ^2^ − 2*ab* + *b* ^2^ = (*a* − *b*)^2^ is positive for any distinct positive values of *a* and *b*, but it is not manifestly positive.) A *rational function* or *rational expression* is the ratio of two polynomials and is itself manifestly positive if both its numerator and denominator polynomials are manifestly positive.

The algebra that gives rise to manifestly positive polynomials is controlled by appropriate subgraphs of *G*, described in the classical Matrix-Tree theorem (MTT), which goes back to 19th century work on electrical circuits (Kirchhoff, 1847; Mirzaev and Gunawardena, 2013); the manifest positivity is exactly what is required for parametric dependence in biology. Steady-state probabilities thereby emerge as manifestly positive rational functions of the edge labels (Eq. 4). This representation has proved very useful in giving mathematical access to steady states (Nam et al., 2022).

An important feature of this rational expression for steady-state probabilities is that it holds for systems that do not necessarily reach a steady state of thermodynamic equilibrium. Briefly, graphs that can reach thermodynamic equilibrium must be *reversible*, so that, given any edge *i* → *j*, there is an edge *j* → *i* that represents the reverse process, and must satisfy the *cycle condition*: the product of the label ratios along any cycle of reversible edges is always 1 (Nam et al., 2022, §4). The cycle condition is equivalent to *detailed balance* or *microscopic reversibility*. In this case, a considerable simplification can be made in describing steady-state probabilities and the resulting expressions turn out to be equivalent to those of equilibrium statistical mechanics (Nam et al., 2022, §4). One great advantage of the linear framework is that it provides a restricted context in which non-equilibrium statistical mechanics can be exactly solved in rational algebraic terms. The functional significance of energy expenditure is a very interesting problem in cellular information processing (Estrada et al., 2016) but lies outside the scope of the present paper. We will mention some of the questions that arise in the Discussion.

A distinguishing feature of the linear framework is that the graph is treated, not just as a description or as a vehicle for doing Matrix-Tree calculations, but as a mathematical entity in its own right, in terms of which general theorems can be formulated. The graph provides a rigorous language in which salient biological features can be precisely expressed while others can be left largely unspecified, thereby allowing some general principles to emerge from behind the overwhelming molecular complexity that is ever present. Among the areas for which this approach has yielded insights are input-output responses (Wong et al., 2018; Yordanov and Stelling, 2018), post-translational modifications (Dasgupta et al., 2014; Nam et al., 2020), allostery (Biddle et al., 2021) and gene regulation (Estrada et al., 2016; Biddle et al., 2019).

Since the initial development of the linear framework, we had long thought that only steady states could be expressed as rational functions of the edge labels. However, as we will show here, important properties of the transient regime, such as first-passage times, can also be calculated as rational functions of the edge labels. The capability to analyse transient behaviour using graph-theoretic methods is particularly welcome because real-time and single-molecule experimental methods are finally giving access to the transient regime within living cells (Kleine Borgmann et al., 2013; Liao et al., 2015; Jones et al., 2017; Loffreda et al., 2017; Chen et al., 2018; Dufourt et al., 2018; Mir et al., 2018; Volkov et al., 2018; Nandan et al., 2022). Much of our understanding of biochemical behaviour has relied on steady-state assumptions, which are not always explicitly stated. The rich complexity of transient behaviours which are beginning to emerge suggests that the time is ripe to develop a more fundamental understanding of the kinds of biochemical computations and information processing that can be achieved transiently. For this, the mathematical methods described here may be of some value.

## 2 Results

### 2.1 Steady states and spanning trees

As preparation for discussing first-passage times, we briefly explain how steady-state probabilities are calculated in terms of the graph; see (Nam et al., 2022, §2) for more details. If we have a graph *G*, we noted in the Introduction that the steady state, *u* ^*∞*^(*G*), satisfies $L\left(G\right)\cdot u^{\infty }\left(G\right)=0$ , so that, in linear algebra terms, *u* ^*∞*^(*G*) lies by definition in the *kernel* of the Laplacian matrix: $u^{\infty }\left(G\right)\in \ker L\left(G\right)$ . If *G* is *strongly connected*—i.e., if, for any pair of distinct vertices *i* and *j*, there is a directed path of edges from *i* to *j*—then this kernel is one-dimensional (Gunawardena, 2012),

$$\dim\;\ker L\left(G\right)=1.$$ (3)

(The structure of kerL(G) is well understood for non-strongly connected graphs (Mirzaev and Gunawardena, 2013). We will not need this for steady states but we will encounter non-strong connectivity when discussing first-passage times in the next section.) Eq. 3 means that if z∈kerL(G) is any nonzero vector, then any other vector in the kernel, such as *u* ^*∞*^(*G*), is a scalar multiple of *z*: *u* ^*∞*^(*G*) = *λz*, for some number *λ*.

The classical Matrix-Tree theorem (MTT) yields a formula for a canonical basis vector, ρ(G)∈kerL(G) . We will describe this formula shortly but note first that, as just mentioned, *u* ^*∞*^(*G*) must be a scalar multiple of *ρ*(*G*), so that $u_{i}^{\infty }\left(G\right)=\lambda \rho_{i}\left(G\right)$ for *i* = 1, …, *N*. Using the conservation law in Eq. 2 and recalling that *u*_tot_ = 1 for probabilities, *λ* may be removed by normalising, so that,

$$u_{i}^{\infty }\left(G\right)=\frac{\rho_{i}\left(G\right)}{\rho_{1}\left(G\right)+\cdots +\rho_{N}\left(G\right)}.$$ (4)

We need some terminology to explain how *ρ*(*G*) is determined from *G*. A *spanning forest*, *F*, of *G* is a subgraph that contains all vertices in *G* (“spanning”), lacks cycles when edge directions are ignored (“forest”), and has at most one outgoing edge from each vertex. The vertices with no outgoing edges are called the *roots* of *F*. If *F* has only one root, it is called a *spanning tree*. A forest consists of separate trees, although the forest is upside down, with each tree ascending to its root. Given any non-empty subset of vertices, *∅* ≠ *U* ⊆{1, *…*, *N*}, let Φ_*U*_(*G*) denote the set of spanning forests of *G* that are rooted at *U*. Finally, given any subgraph *H* of *G*, let *w*(*H*) denote the product of all the edge labels in *H*: *w*(*H*) = *∏*_*i*→*j*∈*H*_ *ℓ*(*i* → *j*). As a matter of convention, if *H* has no edges, then *w*(*H*) = 1. Then, *ρ*_*i*_(*G*) is obtained by summing *w*(*F*) over all spanning trees *F* of *G* that are rooted at *i*,

$$\rho_{i}\left(G\right)=\underset{F\in \Phi_{\left\{i\right\}}\left(G\right)}{\sum }w\left(F\right).$$ (5)

**FIGURE 2:**
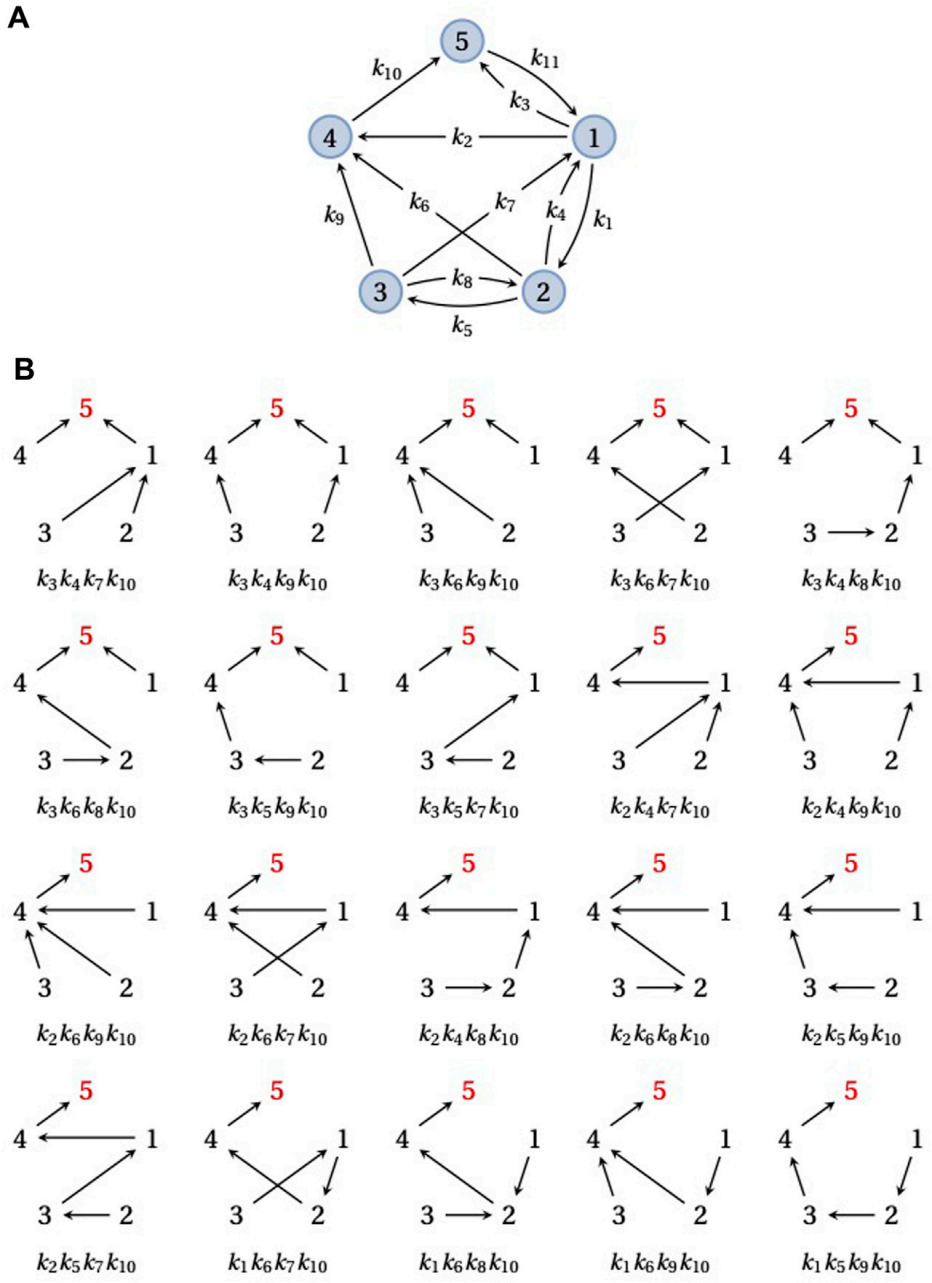
Spanning trees and steady-state probabilities. **(A)** An example graph, *G*, on five vertices, $\left\{1,\ldots ,5\right\}$ , with 11 edges, labeled *k*_1_,*…*, *k*_11_. *G* is strongly connected. **(B)** The 20 spanning trees of *G* rooted at vertex 5 (red), each with its corresponding monomial product of edge labels. The sum of these 20 edge label products gives *ρ*_5_(*G*) in Eq. 5.

*ρ*_*i*_(*G*) is a manifestly positive polynomial in the edge labels, with each *w*(*F*) being a monomial with coefficient +1. The steady-state probabilities, *u* ^*∞*^(*G*), can be recovered from *ρ*_*i*_(*G*) by using Eq. 4. Figure 2 illustrates this calculation for an example graph with five vertices and *i* = 5. Spanning trees are sufficient to calculate steady-state probabilities in Eq. 5 but spanning forests are also needed for the transient quantities considered below (Eqs. 6, 7).

Eq. 5 is a consequence of the classical MTT. The MTT is one of a family of theorems that describe the relationship between the minors of L(G) and spanning forests of *G*. The details of how Eq. 5 arises from the MTT, along with a statement and proof of the MTT itself, are given in Mirzaev and Gunawardena (2013).

Since a strongly connected graph contains at least one directed path from each vertex to every other vertex, there is always at least one spanning tree rooted at each vertex. Therefore, the right-hand side of Eq. 5 is never empty and has at least one term for any choice of *i*. However, the number of rooted spanning trees may depend on the vertex: in Figure 2, there are 20 spanning trees rooted at vertex 5 but the reader can check that there is only one spanning tree rooted at vertex 3. The size of *ρ*_*i*_(*G*) can vary markedly with *i*, depending on the structure of *G*.

It follows from Eq. 4 that *u* ^*∞*^(*G*) is a manifestly positive rational function of the labels and is also always nonzero, irrespective of the values of the labels. It is well known in probability theory that the steady-state probabilities of a Markov process are always positive when the corresponding graph is strongly connected, and here we not only see why this is so but also how to calculate these probabilities in terms of the transition rates.

Manifest positivity is what we would want for a formula that yields a steady-state probability. It is a striking fact that many well-known mathematical formulas of molecular biology, such as those of Michaelis–Menten and King–Altman in enzyme kinetics, Monod–Wyman–Changeux and Koshland–Némethy–Filmer in protein allostery and Ackers–Johnson–Shea in gene regulation, all have the structure of manifestly positive rational functions. However, they are typically derived in entirely different ways. In fact, all these rational functions can be shown to arise from Eqs. 4, 5 applied to appropriate linear framework graphs (Gunawardena, 2012; Wong et al., 2018; Nam et al., 2022), thereby revealing a surprising mathematical unity underlying the complexity of molecular biology.

### 2.2 First-passage times and spanning forests

We turn now from the steady state to the transient regime and specifically to *first-passage times* (FPTs) (Iyer-Biswas and Zilman, 2016). Given a graph *G*, the FPT from one vertex, *i*, to a distinct target vertex, *j* ≠ *i*, is the random variable for the time it takes the underlying Markov process, *X*(*t*), to reach *j* for the first time when starting from *i*. Formally,

$$\Theta_{i,j}\left(G\right)=\inf\left\{t>0\;:\;X\left(t\right)=j|X\left(0\right)=i\right\}.$$

Of interest are the mean and higher moments of the FPT distribution. *Recurrence times* for the process returning to *i* after leaving *i* can be treated similarly, as can FPTs for reaching a subset of target states from a distinct subset of initial states, but we will leave these refinements aside so as not to complicate the discussion.

For the kinds of stochastic molecular systems considered here, FPTs have been used to quantify several properties: the completion time of an enzymatic turnover (Fisher and Kolomeisky, 1999; Kou et al., 2005; Shaevitz et al., 2005; Kolomeisky and Fisher, 2007; Chemla et al., 2008; Garai et al., 2009; Bel et al., 2010; Moffitt et al., 2010; Cao, 2011; Moffitt and Bustamante, 2014); the speed with which an enzyme can discriminate between correct and incorrect substrates (Banerjee et al., 2017; Cui and Mehta, 2018; Mallory et al., 2019); the statistical structure of transcriptional bursting (Lammers et al., 2020); and the time by which a regulated molecule crosses an abundance threshold (Co et al., 2017; Ghusinga et al., 2017; Gupta et al., 2018). We briefly discuss two examples by way of motivation before proceeding to the technical details.

The development of single-molecule techniques for visualising transcription in live cells (Fukaya et al., 2016; Dufourt et al., 2018) has revealed that transcription is often characterised by transient “bursts” of mRNA expression interspersed by periods of inactivity. Efforts to explain how such bursting arises have focussed on stochastic transitions between transcriptionally active and inactive states in a Markovian setting (Peccoud and Ycart, 1995; Lammers et al., 2020). In active states, successive mRNAs are produced in a burst, which is terminated when the system makes a transition to an inactive state. The FPT to reach an active state from an inactive one provides an estimate of the time between bursts, which can be measured experimentally. As noted by Lammers et al. (2020), comparing the distributions of such FPTs offers a sensitive means to discriminate between different gene regulatory models.

FPTs have also been used to quantify the time at which a regulated molecule reaches a specific abundance threshold (Co et al., 2017; Ghusinga et al., 2017; Gupta et al., 2018). An example of this type of system is bacterial lysis by phage *λ*. Upon infecting *Escherichia coli*, phage *λ* expresses a protein, holin S105, that accumulates in the inner cell membrane until a threshold concentration is reached, at which point the holin molecules abruptly initiate lysis by puncturing the membrane with large irregular holes (White et al., 2010). Various other cellular processes, such as bacterial sporulation (Piggot and Hilbert, 2004), cell cycle progression (Liu et al., 2015) and cell migration during development (Gupta et al., 2018), rely on similar thresholding mechanisms. The FPT analysis undertaken by Ghusinga et al. (2017) shows the impact of different regulatory strategies on the variance in the FPT to reach the threshold and gives insight into the regulatory mechanism of bacterial lysis.

Despite their broad usefulness in biology, FPTs have often been calculated by numerical simulations (Lammers et al., 2020) or by analytical methods that rely on the special structure of the model (Ghusinga et al., 2017). We describe here a systematic graph-theoretic scheme, similar to that in Eq. 5, by which the moments of the FPT distribution can be expressed as rational functions of the edge labels.

**FIGURE 3:**
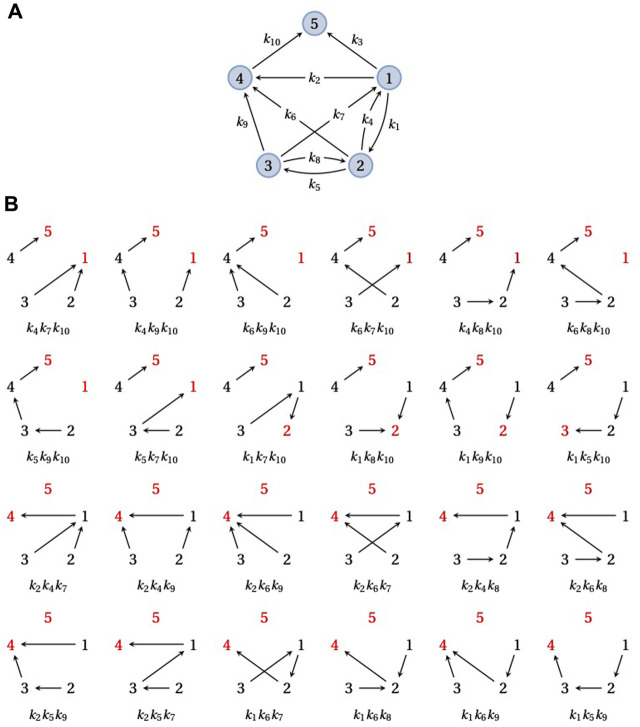
Spanning forests and FPTs. **(A)** An example graph, *G*, obtained by taking the graph in Figure 2A and removing the outgoing edge from vertex 5. *G* has a single terminal SCC containing the single vertex 5. **(B)** The 24 doubly-rooted spanning forests of *G* in which 5 is a root (red font) and there is a path from 1 to the other root (also in red font), each with its corresponding product of edge labels. The sum of these 24 edge label products is equal to the numerator of $\tau_{1,5}^{\left(1\right)}\left(G\right)$ in Eq. 6.

Since Θ_*i*,*j*_(*G*) measures the time taken by *X*(*t*) to reach *j* from *i* for the first time, the distribution of Θ_*i*,*j*_(*G*) does not depend on the outgoing edges from *j* or their labels. Therefore, one can remove from *G* the edges leaving *j* without affecting the distribution of Θ_*i*,*j*_(*G*). For example, the distribution of Θ_*i*,5_(*G*) is the same for the strongly connected graph in Figure 2A and for the graph in Figure 3A, which is formed by removing the edges leaving 5 from the graph in Figure 2A. In consequence, it is convenient when working with FPTs to deal with graphs that may not be strongly connected, for which some additional terminology is helpful.

A graph *G* always has a unique decomposition into *strongly connected components* (SCCs), which can be thought of as the maximal strongly connected subgraphs; see Mirzaev and Gunawardena (2013) for the full details. The directed edges which leave these SCCs give rise to a *partial order* on the set of SCCs. Those SCCs which are maximal in the partial order are called *terminal*. For example, the graph in Figure 2A is strongly connected and therefore has only a single SCC, but if the edge 5 → 1 is removed, to yield the graph in Figure 3A, this graph has 3 SCCs in the partial order {1, 2, 3}⪯{4}⪯{5}. Let us consider the special case where *G* has a unique terminal SCC that contains just one vertex, say, *q* ∈ {1, *…*, *N*}, like the graph in Figure 3A. This is what happens upon removal of the edges leaving a vertex, *q*, in a strongly connected graph, as in Figure 2A: *q* forms a unique terminal SCC, {*q*}, with only one vertex. If the underlying Markov process *X*(*t*) starts from any other vertex, say *i*, then the probability that *X*(*t*) eventually reaches *q* is 1. There may, of course, be trajectories of the process along which *q* is never reached but these form a set of probability zero.

We need just a bit more notation. The quantities we want to calculate are the *k*th moments of the probability distribution of the FPT from *i* to *q*,

$$\tau_{i,q}^{\left(k\right)}\left(G\right)=\left\langle \;\Theta_{i,q}{\left(G\right)}^{k}\;\right\rangle ,$$

where ⟨ − ⟩ denotes the average over the underlying sample space of trajectories. Let I denote the subset of non-terminal vertices, I={1,…,N}\{q} . Given any non-empty subset of vertices, *∅* ≠ *U* ⊂ {1, *…*, *N*}, and vertices *j* ∈ {1, *…*, *N*} and *r* ∈ *U*, let Φ_*U*:*j*⇝*r*_(*G*) denote the set of spanning forests of *G* that are rooted at *U* and contain a directed path of edges from *j* to the root *r*, specified by *j* ⇝ *r*. By convention, there is always a (trivial) directed path from any vertex to itself, so that *r* ⇝ *r*. Then, for the mean FPT, we have (Nam and Gunawardena, 2023),

$$\tau_{i,q}^{\left(1\right)}\left(G\right)=\frac{\sum_{j\in I}\sum_{F\in \Phi_{\left\{j,q\right\}:i⇝j}\left(G\right)}w\left(F\right)}{\sum_{F\in \Phi_{\left\{q\right\}}\left(G\right)}w\left(F\right)}.$$ (6)

The numerator in Eq. 6 runs over all doubly-rooted spanning forests of *G* in which *q* is one root and there is a directed path of edges from *i* to the other root. Figure 3B demonstrates this calculation for the graph in Figure 3A. The denominator in Eq. 6 runs over all spanning trees of *G* rooted at *q* and is similar in that respect to the right-hand side of Eq. 5.

The combinatorics become more complicated for the higher moments of Θ_*i*,*q*_(*G*). Choose *k*-tuples of non-terminal vertices,

$$\left(j_{1},\ldots ,j_{k}\right)\in \underset{k\text{ times}}{\underbrace{I\times \cdots \times I}},$$

and set *j*_0_ = *i*. Then, for the *k*th moment, we have (Nam and Gunawardena, 2023),

$$\tau_{i,q}^{\left(k\right)}\left(G\right)=\frac{k!\sum_{\left(j_{1},\ldots ,j_{k}\right)}\left(\overset{k}{\underset{u=1}{\prod }}\left(\sum_{F\in \Phi_{\left\{j_{u},q\right\}:j_{u-1}⇝j_{u}}\left(G\right)}w\left(F\right)\right)\right)}{{\left(\sum_{F\in \Phi_{\left\{q\right\}}\left(G\right)}w\left(F\right)\right)}^{k}}.$$ (7)

The product in the numerator of Eq. 7 again involves doubly-rooted spanning forests, in which *q* is one of the roots and the other root shifts along the *k*-tuple from *j*_1_ to *j*_*k*_, with *j*_*u*−1_ having a directed path to *j*_*u*_ as *u* runs from 1 to *k*. Eq. 7 reduces to Eq. 6 when *k* = 1.

Note that a spanning forest, or the special case of a spanning tree, that has *q* as a root cannot include any outgoing edge from *q*. Hence, the spanning forests or trees with *q* = 5 as a root are the same for the strongly connected graph in Figure 2A as for the graph in Figure 3A, in which {*q*} has become the unique terminal SCC by removing the edges that leave *q*. Accordingly, both the numerator and denominator in Eqs. 6, 7 give the same result for *q* = 5 in either graph. This is the graph-theoretic consequence of the fact, mentioned above, that the probability distribution of Θ_*i*,5_(*G*) is the same for the graphs in Figure 2A and Figure 3A.

Eq. 7 and, by specialisation, Eq. 6 can be derived, after some manipulations, from the All-Minors Matrix-Tree theorem, a more recent generalisation of the classical MTT (Nam and Gunawardena, 2023).

As a sanity check on Eq. 7, we note that if *G* has *N* vertices, then any spanning forest with *r* roots has *N* − *r* edges, as can be checked for the examples in Figure 2B and Figure 3B. It follows from Eq. 7 that $\tau_{i,q}^{\left(k\right)}\left(G\right)$ has dimensions of (time)^*k*^, as expected for the *k*th moment of an FPT.

Let us see what Eq. 7 tells us for the graph *G* consisting of just two vertices, 1 and 2, with *ℓ*(1 → 2) = *a* and *ℓ*(2 → 1) = *b*. If we consider $\tau_{1,2}^{\left(k\right)}\left(G\right)$ , then, for the denominator of Eq. 7, we need the spanning trees rooted at 2, given by Φ_{2}_(*G*). There is only one such tree *F*, for which *w*(*F*) = *a*. As for the numerator, we need the spanning forests rooted at *j*_*u*_ and 2, given by $\Phi_{\left\{j_{u},2\right\}:j_{u-1}⇝j_{u}}\left(G\right)$ . Since the roots have to be distinct, the only possibility is that *j*_*u*_ = 1. But then the only forest, *F*, with these roots has just these vertices and no edges. Recalling the convention for what happens when there are no edges, we find that *w*(*F*) = 1. It follows that Eq. 7 collapses to the simple conclusion that

$$\tau_{1,2}^{\left(k\right)\left(G\right)}=\frac{k!}{a^{k}}.$$

In particular, the mean FPT is 1/*a* and the variance, which is $\tau_{1,2}^{\left(2\right)}\left(G\right)-{\left(\tau_{1,2}^{\left(1\right)}\left(G\right)\right)}^{2}$ , is 1/*a* ^2^. Only the rate *a* is relevant, as we would expect, since the rate *b* is the label on an edge that leaves the target vertex. Because this example is so simple, the moments of the FPT distribution can be readily calculated without the paraphernalia of Eq. 7. The case of a longer pipeline of vertices is more demanding, as we will see below (Figure 5).

Eq. 7 gives a general and systematic method to calculate FPTs from the linear framework graph associated with a Markov process. It can be used to calculate exact formulas in simple graphs and to avoid estimating FPT moments by cumbersome numerical simulations of the Markov process. The combinatorics rapidly become formidable as the graph becomes larger or less symmetric, as is perhaps already evident in Figure 2B and Figure 3B. The broader value of Eq. 7 is that it reveals the mathematical structure of the FPT moments as manifestly positive rational functions of the edge labels. This can often be informative in its own right, as we will see in discussing enzyme kinetics below. We will say more about ways of dealing with the combinatorial complexity in the Discussion.

### 2.3 Splitting probabilities and conditional FPTs

In the previous section, we considered the FPT distribution from a given vertex *i* to a single target vertex. It is, however, often the case that there are several target vertices and one wants to know the probability of reaching a particular target vertex or the FPT to that vertex conditioned on the Markov process actually reaching it. (If target vertices lie in different SCCs that are not related in the partial order, then a trajectory that reaches one target can never reach any other target, so that the mean FPT to each target becomes infinite. Conditioning on reaching the target is therefore essential.) Let us suppose, therefore, that *G* is a graph with one or more terminal SCCs, each of which consists of a single vertex. Let T⊂{1,…,N} be the subset consisting of these terminal vertices. Given *i* ∈ {1, *…*, *N*} and q∈T , define the *splitting probability from* *i* *to* *q*, denoted *π*_*i*,*q*_(*G*), to be the probability that the underlying Markov process, when started from *i*, eventually reaches *q*, as opposed to any other terminal vertex. Then we have (Nam and Gunawardena, 2023),

$$\pi_{i,q}\left(G\right)=\frac{\sum_{F\in \Phi_{T:i⇝q}\left(G\right)}w\left(F\right)}{\sum_{F\in \Phi_{T}\left(G\right)}w\left(F\right)}.$$ (8)

**FIGURE 4:**
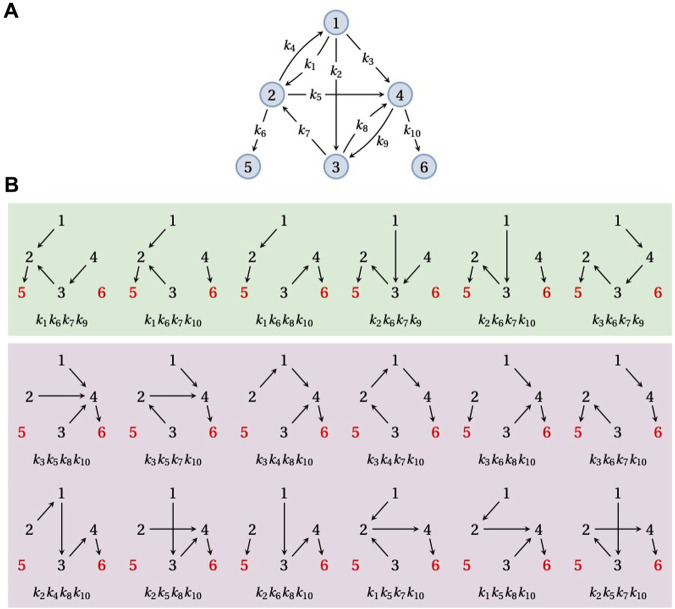
Splitting probabilities. **(A)** An example graph, *G*, on six vertices, $\left\{1,\ldots ,6\right\}$ , with three SCCs. The partial order is given by {1, 2, 3, 4}⪯{5} and {1, 2, 3, 4}⪯{6}, with {5} and {6} being the two terminal SCCs. **(B)** The 18 spanning forests of *G* rooted at vertices 5 and 6 (red font), with those containing a path from 1 to 5 in the green box and those containing a path from 1 to 6 in the purple box. Each spanning forest is shown with its corresponding product of edge labels. The sum of all 18 edge label products is equal to the denominator of *π*_1,5_(*G*) in Eq. 8; the sum of the six edge label products in the green box is equal to the numerator of *π*_1,5_(*G*) in Eq. 8.

The denominator in Eq. 8 runs over all spanning forests of *G* rooted at T , and the numerator runs over the subset of those spanning forests in which there is a directed path of edges from *i* to the root *q*. Accordingly, the right-hand side of Eq. 8 must lie between 0 and 1, as expected for a probability. If i∈T and *i* ≠ *q*, then there is no directed path from *i* to *q* and so Eq. 8 gives 0, while if *i* = *q*, then every spanning forest has a (trivial) path of directed edges from *i* to *q* and so Eq. 8 gives 1. If *G* contains only one terminal vertex, then every spanning forest of *G* rooted at T={q} has a path of directed edges from *i* to *q*, and so Eq. 8 again gives 1. Figure 4 illustrates the calculation of the splitting probability from *i* = 1 to *q* = 5 on a six-vertex graph with two terminal vertices, 5 and 6.

Let us turn now to the conditional FPT for reaching a particular target vertex, q∈T , from the vertex i∈I , where, as before, I is the subset of non-terminal vertices, I={1,…,N}\T . For the mean conditional FPT from i∈I to q∈T , denoted by $\chi_{i,q}^{\left(1\right)}\left(G\right)$ , we find that (Nam and Gunawardena, 2023),

$$\chi_{i,q}^{\left(1\right)}\left(G\right)=\frac{\sum_{j\in I}\left(\sum_{F\in \Phi_{T\cup \left\{j\right\}:i⇝j}\left(G\right)}w\left(F\right)\right)\left(\sum_{F\in \Phi_{T:j⇝q}\left(G\right)}w\left(F\right)\right)}{\left(\sum_{F\in \Phi_{T:i⇝q}\left(G\right)}w\left(F\right)\right)\left(\sum_{F\in \Phi_{T}\left(G\right)}w\left(F\right)\right)}.$$ (9)

If there is only one terminal vertex, so that T={q} , then the mean conditional FPT, $\chi_{i,q}^{\left(1\right)}\left(G\right)$ , as given by Eq. 9, is equal to the mean FPT, $\tau_{i,q}^{\left(1\right)}\left(G\right)$ , as given by Eq. 6. Formulas for the higher moments of the conditional FPT can be obtained in a similar way.

Evidently, the unconditional mean FPT to reach any terminal vertex in T from *i*, denoted $\psi_{i}^{\left(1\right)}\left(G\right)$ , is now given by,

$$\psi_{i}^{\left(1\right)}\left(G\right)=\underset{p\in T}{\sum }\pi_{i,p}\left(G\right)\;\chi_{i,p}^{\left(1\right)}\left(G\right).$$

Combining Eqs. 8, 9, we can show that this mean FPT can also be expressed in terms of the spanning forests of *G*, as

$$\psi_{i}^{\left(1\right)}\left(G\right)=\frac{\sum_{j\in I}\left(\sum_{F\in \Phi_{T\cup \left\{j\right\}:i⇝j}\left(G\right)}w\left(F\right)\right)}{\left(\sum_{F\in \Phi_{T}\left(G\right)}w\left(F\right)\right)},$$ (10)

which specialises to Eq. 6 when there is only a single terminal vertex.

Splitting probabilities and conditional FPTs have not been as widely used as have the unconditional FPTs described in the previous section. This reflects the relatively simple models that have been formulated so far in the literature. However, as we have shown here, there is no greater difficulty in dealing with these more complex quantities, at least within the graph-theoretic approach that we have outlined here. All the quantities we have considered are manifestly positive rational functions of the edge labels. This mathematical accessibility should allow deeper analysis of transient stochastic properties.

### 2.4 Single-molecule enzyme kinetics

Single-molecule experimental methods have given unprecedented access to the stochastic kinetics of individual enzymes and have stimulated the development of theoretical models to account for the resulting data. This literature offers a convenient setting to illustrate the ideas introduced above.

**FIGURE 5:**
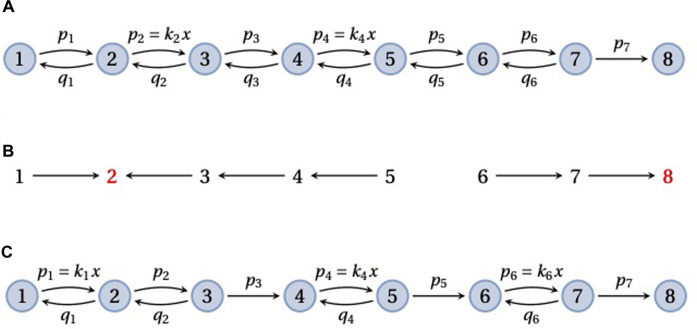
Pipeline graphs. **(A)** A pipeline graph on 8 vertices that consists of a single reversible block, with substrate binding with concentration *x* at the edges 2 → 3 and 4 → 5, followed by a single irreversible transition, 7 → 8. **(B)** The spanning forest *F*(2, 6, 8), in the notation described in the text, for the graph in panel **A**. The two roots, 2 and 8, are in red font. **(C)** A pipeline graph with three reversible blocks, in each of which the substrate binds once. As explained in the text, the mean FPT, $\tau_{1,8}^{\left(1\right)}\left(G\right)$ , has a reciprocal Michaelis–Menten dependence on the substrate concentration, *x*, as in Eq. 15.

A frequently used model in enzyme kinetics corresponds to a *pipeline* graph (Figure 5) (Fisher and Kolomeisky, 1999; Kou et al., 2005; Kolomeisky and Fisher, 2007; Chemla et al., 2008; Garai et al., 2009; Moffitt et al., 2010; Moffitt and Bustamante, 2014). Such a graph consists of vertices 1, *…*, *N*, representing different conformations of the enzyme, with nearest-neighbour transitions, *i* → *i* + 1 or *i* → *i* − 1. Substrate may bind at any forward transition, *i* → *i* + 1, so that *ℓ*(*i* → *i* + 1) incurs a concentration term that we will denote by *x*, and binding is assumed to be reversible, so that *i* + 1 → *i*. The final transition, *N* − 1 → *N*, is usually treated as an irreversible catalytic step, with the enzyme returning to its initial conformation, so that vertex *N* corresponds to vertex 1 in the next enzymatic cycle. A pipeline may be thought of as partitioned into reversible “blocks” that are separated by sequences of irreversible transitions. Figures 5A, C show pipeline graphs with 1 and 3 reversible blocks, respectively.

The mean FPT for reaching vertex *N* from vertex 1 is a measure of the enzyme’s completion time. Bustamante and colleagues have emphasised how the substrate dependence of $\tau_{1,N}^{\left(1\right)}\left(G\right)$ and $\tau_{1,N}^{\left(2\right)}\left(G\right)$ contains information about the enzyme mechanism, and they have built on previous studies (Derrida, 1983) to analyse this theoretically (Moffitt et al., 2010). This amounts to studying $\tau_{1,N}^{\left(1\right)}\left(G\right)$ and $\tau_{1,N}^{\left(2\right)}\left(G\right)$ as functions of *x*, which falls directly into the scope of the results described above. We will show how the graph-theoretic methods introduced here provide a straightforward way to recover some of these previous findings. We do not intend to be exhaustive and there is much more of interest in the cited references. We hope, rather, to show the advantages of the graph-theoretic approach over the variety of approaches used previously, such as recursive solution of the master equation (Derrida, 1983) or Fourier transformation and determinants (Chemla et al., 2008).

Consider first a pipeline graph, *G*, with a single reversible block consisting of the vertices 1, *…*, *N* − 1 and recall Eq. 6 for the mean FPT, where the terminal vertex is *q* = *N*. An example is shown in Figure 5A with the notation that we will use for the edge labels, *ℓ*(*i* → *i* + 1) = *p*_*i*_ and *ℓ*(*i* + 1 → *i*) = *q*_*i*_. It is evident that there is only a single spanning tree, *T* ∈ Φ_{*N*}_(*G*), consisting of all the forward edges, so that *w*(*T*) = *p*_1_⋯*p*_*N*−1_. This gives the denominator of $\tau_{1,N}^{\left(1\right)}\left(G\right)$ . As for the doubly-rooted spanning forests of Φ_{*j*,*N*}_(*G*) in the numerator, they can be indexed as *F*(*j*, *k*, *N*), where *j* < *k* ≤ *N* and *k* is the vertex with the smallest index that has a directed path to the root *N* (Figure 5B). Furthermore, each such forest has a directed path from 1 to the root *j*, so that Φ_{*j*,*N*}:1⇝*j*_(*G*) = Φ_{*j*,*N*}_(*G*). We see from the labels in Figure 5B that

$$w\left(F\left(j,k,N\right)\right)=p_{1}\cdots p_{j-1}q_{j}\cdots q_{k-2}p_{k}\cdots p_{N-1},$$ (11)

where the “missing” label, between vertices *k* − 1 and *k*, corresponds to the gap between the tree rooted at *j* and the tree rooted at *N* in the forest. If we divide by the denominator, we see that each spanning forest *F*(*j*, *k*, *N*) contributes a rational function of the labels that we may write in the form,

$$\frac{w\left(F\left(j,k,N\right)\right)}{w\left(T\right)}=\frac{1}{p_{j}}\overset{k-2}{\underset{u=j}{\prod }}\frac{q_{u}}{p_{u+1}}.$$

The spanning forests in Φ_{*j*,*N*}_(*G*) therefore contribute the sum,

$$\frac{\sum_{F\in \Phi_{\left\{j,N\right\}:1⇝j}\left(G\right)}w\left(F\right)}{w\left(T\right)}=\frac{\Delta \left(j,N\right)}{p_{j}},$$

where,

$$\Delta \left(j,N\right)=\overset{N}{\underset{k=j+1}{\sum }}\left(\overset{k-2}{\underset{u=j}{\prod }}\frac{q_{u}}{p_{u+1}}\right).$$ (12)

Note that, in Eq. 12, the empty product for *k* = *j* + 1 is by convention taken to be 1. It follows from Eq. 6 that the enzyme completion time is given by,

$$\tau_{1,N}^{\left(1\right)}\left(G\right)=\overset{N-1}{\underset{j=1}{\sum }}\frac{\Delta \left(j,N\right)}{p_{j}}.$$ (13)

With some notational translation, Eq. 13 can be seen to be the same as (Moffitt et al., 2010, Eq. S2). The quantity Δ(*j*, *N*) in Eq. 12 first appears in Derrida’s derivation of the velocity and diffusion constant of a Markov particle on a periodic pipeline (Derrida, 1983, Eq. 24); Δ(*j*, *N*) = Γ(*j* + 1, *N* − 1), where Γ is the quantity defined in Eq. S3 of Moffitt et al. (2010). The calculation above, using the general formula for the mean FPT in Eq. 6, is hopefully more transparent.

Suppose now that substrate binds at *s* forward transitions in the pipeline graph, with concentration *x*. We will refer to terms other than *x* in the edge labels as “kinetic parameters,” which thereby include both simple rates and on-rates. Since we can exclude the final catalytic transition from substrate binding, it follows that 1 ≤ *s* ≤ *N* − 2. Eq. 11 then shows that the enzyme completion time has the following structure as a rational algebraic function of *x*,

$$\tau_{1,N}^{\left(1\right)}\left(G\right)=\frac{a_{0}+a_{1}x+\cdots +a_{s}x^{s}}{bx^{s}}.$$ (14)

Here, the coefficients *a*_0_, *…*, *a*_*s*_ and *b* are all manifestly positive polynomials in the kinetic parameters. In particular, the forest *F*(*N* − 1, *N*, *N*) includes all the substrate-binding transitions, which confirms that *a*_*s*_ > 0. If the substrate-binding transitions are specified, these polynomials may be explicitly calculated using Eq. 11. Eq. 14 already provides some insight. In the limit of low substrate, the completion time diverges at an order, 1/*x* ^*s*^, that depends on the number of substrate-binding transitions. In contrast, in the limit of high substrate, the completion time asymptotes to the positive value *a*_*s*_/*b*. If substrate binds at only one transition in the pipeline, so that *s* = 1, then the completion time exhibits a reciprocal Michaelis–Menten form (Kou et al., 2005; Garai et al., 2009; Moffitt et al., 2010; Moffitt and Bustamante, 2014) (Discussion),

$$\tau_{1,N}^{\left(1\right)}\left(G\right)=\frac{a_{0}+a_{1}x}{bx}.$$ (15)

The higher moments of the FPT, as specified by Eq. 7, are more complicated to calculate but the doubly-rooted spanning forests that are needed for the numerator, which are contained in $\Phi_{\left\{j_{u},N\right\}:j_{u-1}⇝j_{u}}\left(G\right)$ , have already been enumerated by the forests *F*(*j*, *k*, *N*) introduced above (Figure 5B). It seems reasonable to conclude from Eq. 7 that $\tau_{1,N}^{\left(k\right)}\left(G\right)$ has a similar rational algebraic structure as shown in Eq. 14 but with a degree of *ks* for both the numerator and the denominator. In particular, if substrate binds at only one transition, so that *s* = 1, the second moment of the FPT is a quadratic rational function (Moffitt et al., 2010).

In their study of the packaging motor for the *φ*29 bacteriophage, Bustamante and colleagues consider a more general pipeline graph, *G*, that consists of multiple reversible blocks separated by single irreversible transitions (Figure 5C) (Moffitt et al., 2010). The packaging motor is a pentameric ring of identical ATPase units that compacts the *φ*29 double-stranded DNA into the assembling viral capsid. It has been found to do this in a burst of four ATP-consuming steps per cycle. ATP hydrolysis during the catalytic step is typically irreversible under physiological conditions and a pipeline with 4 reversible blocks serves as a model for the motor (Moffitt et al., 2010, Figure 4A).

If the Markov process takes an irreversible transition in *G*, it cannot subsequently visit the preceding reversible blocks. Also, every irreversible transition must be taken to reach *N*. Hence, any trajectory that begins at 1 and reaches *N* must take each irreversible transition exactly once. It follows from this that the FPT from 1 to *N* is just the sum of the FPTs for each reversible block considered separately and these FPTs are all independent of each other. Suppose there are *m* reversible blocks which start at the vertices *e*_0_, *e*_1_, …, *e*_*m*−1_, where 1 = *e*_0_ < *e*_1_ < *e*_2_ < ⋯ < *e*_*m*−1_ < *N*. Let *G*_*i*_ be the subgraph consisting of the vertices from *e*_*i*−1_ to *e*_*i*_, which includes the *i*th reversible block and the immediately following irreversible transition. It follows that,

$$\tau_{1,N}^{\left(k\right)}\left(G\right)=\tau_{1,e_{1}}^{\left(k\right)}\left(G_{1}\right)+\tau_{e_{1},e_{2}}^{\left(k\right)}\left(G_{2}\right)+\cdots +\tau_{e_{m-1},N}^{\left(k\right)}\left(G_{m}\right).$$ (16)

If substrate binds at the same number of transitions in each reversible block, then Eq. 7 shows that the $\tau_{e_{i-1},e_{i}}^{\left(k\right)}\left(G_{i}\right)$ all have the same rational algebraic structure with the same degrees in both the numerator and the denominator. It follows from Eq. 16 that $\tau_{1,N}^{\left(k\right)}\left(G\right)$ must also have this same rational algebraic structure. For the case of the *φ*29 packaging motor, ATP binds at only one transition in each reversible block, so the completion time has the reciprocal Michaelis–Menten form of Eq. 15 and the resulting curve may be fitted to the experimental data (Moffitt et al., 2010, Figure 3A). Bustamante and colleagues make use of the reciprocal of the coefficient of variation,

$$n_{\text{min}}=\frac{{\left(\tau_{1,N}^{\left(1\right)}\left(G\right)\right)}^{2}}{\tau_{1,N}^{\left(2\right)}\left(G\right)-{\left(\tau_{1,N}^{\left(1\right)}\left(G\right)\right)}^{2}},$$

which is readily seen from the discussion above to be a quadratic rational function of *x*, and they also fit this curve to the experimental data (Moffitt et al., 2010, Figure 3B). A theorem due to Aldous and Shepp (1987), which is of independent interest, tells us that, for an arbitrary graph with *N* vertices, *n*_min_ < *N*.

An interesting question arises as to whether *n*_min_ itself is also manifestly positive, as might be expected of a coefficient of variation, given that this is true for both $\tau_{1,N}^{\left(1\right)}\left(G\right)$ and $\tau_{1,N}^{\left(2\right)}\left(G\right)$ . A further point made by Moffitt et al. (2010) is that the quadratic structure of *n*_min_ may not be limited to pipeline graphs but may be true also for some graphs with branches and parallel pathways. If so, the graph-theoretic methods described here offer a way to generalise their findings.

## 3 Discussion

We have reviewed here how the graph-theoretic linear framework, as applied to continuous-time Markov processes, can be used to show that the moments of the FPT distribution (Eqs. 6, 7), splitting probabilities (Eq. 8) and conditional mean FPTs (Eq. 9) can be exactly expressed as manifestly positive rational algebraic functions of the edge labels or transition rates. This reveals that not only steady-state probabilities but also transient properties of Markov processes have this same algebraic structure, thereby substantially expanding the mathematical scope of the linear framework.

The formulas given here can be used to obtain closed-form solutions for simple graphs, as we showed for the pipeline graphs used in enzyme kinetics (Eq. 13). However, this is a little misleading because enumeration of spanning forests becomes rapidly intractable as the graph becomes larger or less symmetric. Moreover, as is evident by examining the algebraic terms in Figure 2B and Figure 3B, every label in the graph can appear in the formulas. There is both a combinatorial explosion and a global parametric dependence. These challenges have long been recognised when dealing with steady-state probabilities (Nam et al., 2022), before the transient regime became mathematically accessible, and several strategies have emerged for dealing with them.

First, when properties of interest are treated as functions of substrate concentration, a great deal can be said about the resulting rational algebraic structure, even when it is hard to calculate the coefficients explicitly in terms of the edge labels (Thomson and Gunawardena, 2009; Nam et al., 2022). As we saw with Eq. 14, the algebraic structure for the mean FPT, $\tau_{1,N}^{\left(1\right)}\left(G\right)$ , is highly informative, especially with respect to the limits of low or high concentration, which may also be experimentally accessible. The Michaelis–Menten structure, or its reciprocal in Eq. 15, arises in a remarkably wide range of biological contexts that are far removed from the 3-vertex pipeline graph considered, in effect, by Michaelis and Menten (Michaelis and Menten, 1913). The linear framework allows general theorems to be proved, which characterise many of the contexts in which the Michaelis–Menten structure does appear (Wong et al., 2018). In this respect, the context discussed above, of a pipeline graph with multiple reversible blocks, in which substrate binds once in each block, falls outside the scope of the theorems in Wong et al. (2018). As suggested by Moffitt et al. (2010), it seems plausible that the Michaelis–Menten structure may also arise for more complicated graphs and an interesting problem arises in characterising this new context.

Second, the question of when the Michaelis–Menten structure arises is closely related to whether or not the graph satisfies the cycle condition and can thereby reach a steady state of thermodynamic equilibrium. If it can, there is a necessary and sufficient condition for the emergence of the Michaelis–Menten structure; if it cannot, and the graph reaches a non-equilibrium steady state, then only partial sufficient conditions are known (Wong et al., 2018). Of course, the pipeline example just mentioned cannot reach thermodynamic equilibrium, as it contains irreversible transitions (Figure 5A). If the cycle condition is satisfied, the complexity problem is substantially reduced, insofar as calculating steady-state probabilities is concerned. It is possible to find an alternative basis element to *ρ*(*G*) in kerL(G) (Eq. 5), which is based on paths rather than spanning trees, for which the combinatorial explosion disappears and the parametric dependence becomes local, not global (Nam et al., 2022). It is a very interesting question as to whether transient quantities like FPTs show any similar reduction in complexity for graphs that satisfy the cycle condition.

Aside from the calculational complexity, the thermodynamic issues also have a deep impact on biological function. The role of energy expenditure in force generation or pattern formation has been widely studied (Kolomeisky and Fisher, 2007; Karsenti, 2008) but its significance for cellular information processing has been more elusive (Wong and Gunawardena, 2020). In the latter domain, unlike the two former ones, information processing can take place at thermodynamic equilibrium, for instance, through binding and unbinding. However, there is a limit to how well this can be done, as first pointed out by Hopfield (1974). We have introduced the concept of the *Hopfield barrier*, as the limit to how well a given information processing task can be undertaken by a mechanism that operates at thermodynamic equilibrium (Estrada et al., 2016). For example, the Hill function with Hill coefficient *n* is the universal Hopfield barrier for the sharpness of input-output responses with *n* binding sites for the input (Nam et al., 2022; Martinez-Corral et al., 2023). Another interesting question arises as to whether there are also Hopfield barriers in the transient regime. That is, if a graph satisfies the cycle condition and can reach a steady state of thermodynamic equilibrium, are there limits on the moments of the FPT distribution, $\tau_{i,q}^{\left(k\right)}\left(G\right)$ , which can only be exceeded if energy is expended to break the cycle condition, allowing the system to reach a non-equilibrium steady state?

Third, the algebraic complexity of non-equilibrium steady states can be reorganised to make the complexity more tractable (Çetiner and Gunawardena, 2022). This breakthrough has enabled steady-state calculations to be undertaken that were previously out of reach. It is conceivable that similar kinds of reorganisation may also throw light on the calculation of transient quantities. Finally, a fourth potential approach to overcoming the complexity is to exploit the recursive technique for enumerating spanning forests that was developed by Chebotarev and Agaev (2002). While this technique looks promising, it has yet to be properly exploited.

The methods outlined here bring the FPTs of Markov processes into focus as manifestly positive rational algebraic functions of the transition rates. This gives mathematical access to them in a way that has been lacking in previous treatments, which have not exploited graph theory and the Matrix-Tree theorems. We hope this review will encourage more use of the linear framework in cell and developmental biology. We anticipate that, as we have found for steady states, this exploration will lead to further general principles and mathematical theorems that rise above the molecular complexity that confronts us in biology.

## Data Availability

The original contributions presented in the study are included in the article/Supplementary Material, further inquiries can be directed to the corresponding author.
